# Effect of landiolol on sex-related transcriptomic changes in the myocardium during sepsis

**DOI:** 10.1186/s40635-019-0263-0

**Published:** 2019-08-19

**Authors:** Thi Thom Tran, Calypso Mathieu, Magali Torres, Béatrice Loriod, Linh Thuy Lê, Catherine Nguyen, Monique Bernard, Marc Leone, Nathalie Lalevée

**Affiliations:** 1 Aix-Marseille Univ, INSERM UMR 1090, TAGC, Campus de Luminy, Case 928, 13288 Marseille Cedex 9, France; 2 Aix Marseille Univ, Service d’anesthésie et de réanimation, Hôpital Nord, Assistance Publique Hôpitaux de Marseille, Chemin des Bourrely, 13015 Marseille, France; 3 Aix-Marseille Univ, INSERM UMR 1090, TGML, Marseille, France; 4 Aix-Marseille Univ, CNRS, CRMBM, Marseille, France

**Keywords:** Genes, Dimorphism, Sex, Sepsis, Beta-blocker, Transcriptome

## Abstract

**Objectives:**

The aims of this study are to better understand phenotypic differences between male and female rats during sepsis, to characterise the contribution of the beta1-adrenergic blocker landiolol to septic cardiomyopathy and to determine why landiolol induces divergent effects in males and females.

**Methods:**

The myocardial transcriptional profiles in male and female Wistar rats were assessed after the induction of sepsis by cecal ligation and puncture and addition of landiolol.

**Results:**

Our results showed major differences in the biological processes activated during sepsis in male and female rats. In particular, a significant decrease in processes related to cell organisation, contractile function, ionic transport and phosphoinositide-3-kinase/AKT (PI3K/AKT) signalling was observed only in males. The transcript of ATPase sarcoplasmic/endoplasmic reticulum Ca^2+^ transporting 3 (SERCA3) was sex-differently regulated. In males, landiolol reversed several signalling pathways dysregulated during sepsis. The expression level of genes encoding tubulin alpha 8 (TUBA8) and myosin heavy chain 7B (MYH7) contractile proteins, phosphatase 2 catalytic subunit alpha (PPP2CA), G protein-coupled receptor kinase 5 (GRK5) and A-kinase anchoring protein 6 (AKAP6) returned to their basal levels. In contrast, in females, landiolol had limited effects.

**Conclusion:**

In males, landiolol reversed the expression of many genes that were deregulated in sepsis. Conversely, sepsis-induced deregulation of gene expression was less pronounced in females than in males, and was maintained in the landiolol-treated females. These findings highlight important sex-related differences and confirm previous observations on the important benefit of landiolol intake on cardiac function in male rats.

**Electronic supplementary material:**

The online version of this article (10.1186/s40635-019-0263-0) contains supplementary material, which is available to authorized users.

## Background

The pathophysiology of sepsis involves a complex mix of systemic factors and molecular, metabolic and structural changes in cardiomyocytes [1]. These include the presence of circulating depressant factors, such as endotoxin and nitric oxide [2], pro-inflammatory cytokines [3], myocyte cell death, abnormal cardiac energetics [4, 5], alterations in adrenergic signalling and intra-cellular calcium cycling, impaired electromechanical coupling and mitochondrial dysfunction [6].

The adrenergic system plays a key role in sepsis, and β-adrenergic modulation as a therapeutic intervention needs to be properly understood [7, 8]. β-blockers showed promising results when the treatment was started before the septic insult or after haemodynamic stabilisation [9–11]. In patients with septic shock, the β-blocker esmolol reduced the heart rate, increased the stroke volume and reduced the need for norepinephrine [10]. In experimental models of sepsis, a selective β1-blocker efficiently improved both cardiac and vascular functions and down-regulated inflammatory pathways [12–16]. Differences in transcripts in the β-adrenergic signalling, calcium cycling pathways and impaired electromechanical coupling were linked with an unfavourable outcome [17]. Regarding gene expression, to our knowledge, no large-scale studies have been conducted on the effect of a selective β1-blocker in experimental models of sepsis.

There is increasing evidence of sex-related differences in the cardiovascular system [18, 19], in the response to β-blockers [20, 21] and in the host response to an inflammatory insult [22–25]. In experimental models of sepsis, cardiac performance was more impaired in males as compared with that in females [25, 26]. Male mice exhibited dysregulation of myocardial calcium transporters, in particular the sarcoplasmic reticulum calcium ATPase (SERCA) [27, 28] and female mice developed cardiac dysfunction as a result of cyclic guanosine monophosphate-mediated depression of myofilament function [29].

In our recent study, we showed for the first time sex-related disparities in the response to the β-blocker landiolol in rats with sepsis-induced myocardial dysfunction [26]. This data showed that landiolol improved the in vivo cardiac performance in male rats exposed to cecal ligation and puncture (CLP), whereas it induced deleterious effects in female rats. We confirmed the presence of sex-related differences in inflammatory response, calcium signalling and apoptosis pathways during sepsis [26].

This data raised the question of the origin of sex-related phenotypic differences during sepsis and in response to landiolol. To answer these questions, we conducted a transcriptional study in the septic male and female rat hearts and investigated the biological processes reversed by landiolol infusion.

## Material and methods

### Animals

Male and female Wistar rats aged 9–12 weeks were reused from a recent study [26]. All animal procedures were approved by the Institutional Animal Care Committee of Aix-Marseille University (APAFIS number: 3746-201601221813985). This allowed us to correlate the results of the transcriptomic analysis in this study with the in vivo findings obtained in our earlier study on the cardiac status performance. In addition, it reduced the number of animals used for experimentation [26].

### Experimental protocol

The experimental procedure was described earlier [26]. Briefly, 1 h after CLP, the animals were randomized to receive landiolol (AOP Orphan, Vienna, Austria), diluted in n-saline that did not contain antibiotics and infused at 0.1 mg kg^−1^ min^−1^, or n-saline (10 ml kg^−1^ h^−1^). The infusion volume was similar in all the groups. Buprenorphine (0.05 mg kg^−1^) was used for pain control before surgical procedure. The 43 animals were distributed into six groups: a sham group (males, *n* = 6; females, *n* = 7), a CLP group (males, *n* = 8; females, *n* = 8) and a CLP plus landiolol group (males, *n* = 7; females, *n* = 7). No animals died prematurely from sepsis. At hour 18, in vivo cardiac magnetic resonance imagings (MRIs) were performed followed by ex vivo cardiac function measurement using Langendorff isolated heart preparation. At hour 20, animals were sacrificed and the hearts were frozen. The left ventricle tissues were used for RNA preparation. Three hearts (two CLP male and one sham female) were not frozen due to handling problems. Eight RNA samples were discarded because of their insufficient quality (low RNA integrity number (RIN)) or quantity. Quantification of gene expression was performed on 32 RNA samples from the sham (males, *n* = 5; females, *n* = 5), CLP (males, *n* = 5; females, *n* = 5) and CLP plus landiolol (males, *n* = 6; females, *n* = 6) groups.

### RNA preparation

Total RNA was isolated from the left ventricle using TRIzol reagent according to the manufacturer’s instructions (Invitrogen, California). The concentration of RNA was determined by reading the absorbance at 260 nm using a NanoDrop (ND-1000, Thermo Fischer Scientific, MA, USA). The ratio of A260/280 in all the RNA samples ranged from 1.8 to 2.0. The quality of RNA was confirmed using an Agilent 2100 Bioanalyzer (Agilent Technologies, Germany) with Agilent RNA 6000 Nano Chips. Samples with a RIN higher than 8 were used in a microarray.

### Gene expression measurements

Gene expression was measured using a SurePrint G3 Rat GE8x60K Microarray Kit (Agilent Technologies, CA, USA) containing 62,976 oligonucleotide probes representing 30,003 genes. Total RNAs (200 ng) were labelled with cyanine 3-CTP using a Low RNA Input Linear Amplification Kit according to the manufacturer’s protocol (Agilent Technologies, CA, USA). For each reaction, the cRNA yield and specific activity of cRNA were determined using a NanoDrop ND-1000 spectrophotometer. Only the cRNAs with yields of > 0.9 μg and specific activities > 6.0 pmol of dye per microgram of cRNA were used for hybridisation. The labelled cRNAs were hybridised to microarray slides (eight arrays per slide) following the Agilent One-Color microarray-based gene expression analysis protocol (Agilent Technologies, CA, USA). The slides were scanned (8 × 60 k array slides at 3 μm resolution) using an Agilent DNA microarray scanner (G2505C) and colour setting of an Agilent G3_GX_1. The scanned images were analysed using Feature Extraction Software 10.5 (Agilent).

### Statistical analysis

Statistical evaluation determined that the number of rats required for in vivo experiments to show a 30% increase in SV by landiolol with a power and an alpha risk of 80% and 5%, respectively, was six per group. From the 6–8 animals per group tested in vivo, 5–6 hearts per group were selected as described in the above experimental protocol and used for gene expression quantification. No statistical evaluation was a priori performed but five biological replicates per group are empirically required for common designs [30]. In total, 32 raw data files (five arrays for the sham, five arrays for the CLP and six arrays for the CLP plus landiolol groups, for each sex) were obtained using the Agilent Feature Extraction Software. The raw intensity data were exported as a single Excel spread sheet, and the raw data was converted in log2. Quantile normalisation was then applied to correct for global intensity and dispersion, and an 80% filtering was used to keep only genes expressed over the background noise. This procedure generated 38,223 probes in males and 37,605 probes in females. All the microarray data files are accessible at NCBI’s Gene Expression Omnibus website (accession number: GSE125042). To detect significant variation in gene expression between groups (CLP group vs sham group and CLP plus landiolol group vs CLP group), the significance analysis of microarrays (SAM) method was used, with 10,000 permutations applied with a false discovery rate (FDR) of 1% or 5%. Hierarchical clustering of differentially expressed genes (DEGs) was achieved using the TM4 Microarray Software Suite V4.9 (http://mev.tm4.org) and average linkage clustering metrics. Pearson’s correlation was used to determine the distance. Venn diagrams were generated to quantify sex-related differences in common and specific DEGs in the various groups.

### Functional annotations

For biological interpretation of the gene expression data, enriched functional annotations for the up- and down-regulated genes were identified using the Database for Annotation, Visualization and Integrated Discovery (DAVID) Bioinformatics Resources 6.7 (https://david.ncifcrf.gov). Biological processes, cellular components and Kyoto Encyclopedia of Genes and Genomes (KEGG) pathways were considered significantly enriched when P-Benjamini < 0.05. The KEGG pathways (http://www.genome.jp/kegg/) were also used when no significant biological processes were found in DAVID. Cytoscape was used to design the gene network of CLP genes whose expression was reversed after landiolol administration (http://www.cytoscape.org).

## Results

### Sex-related differential expression of genes in the CLP group and CLP plus landiolol group

**Fig. 1:**
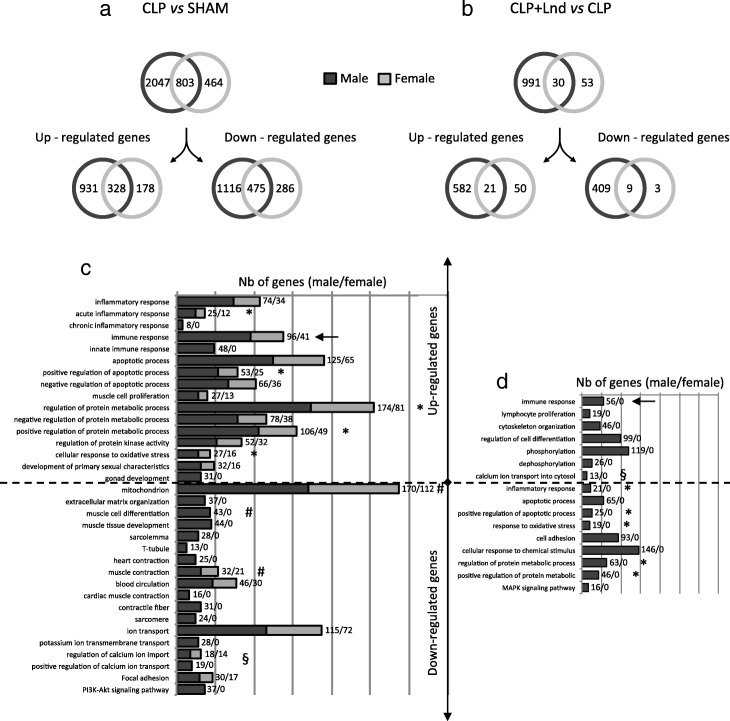
Differentially expressed genes (DEG) in male and female hearts after cecal ligation and puncture and landiolol. DEG were obtained after 2-class significance analysis of microarrays (SAM) 2 classes in TMeV, 10,000 permutations with an FDR < 0.01 for CLP vs sham (**a**) and with an FDR < 0.05 for CLP plus landiolol vs CLP groups (**b**). **a**, **b** Venn diagrams show the total number of DEG and the number of genes significantly up- and down-regulated in CLP (**a**) and CLP plus landiolol groups (**b**). The number of specific genes in females is very low compared to males. **c**, **d** A gene ontology analysis realised with the DAVID Database is reported on graphs in which the most significant biological process (BP)-GO terms for up- and down-regulated genes are indicated for CLP vs sham groups (**c**) and for CLP plus landiolol vs CLP groups (**b**). For all the processes, the number of genes deregulated in males is much greater than in females. Note the total absence of significant biological processes for females after landiolol infusion in (**d**). (*) indicates over-expressed biological processes during CLP that were reversed by landiolol. (§) indicates under-expressed biological processes during CLP that were reversed by landiolol. (#) indicates under-expressed biological processes related to cardiac activity and cardiomyocyte function that were affected during CLP and were not restored by landiolol. (←) shows that the immune response is over-expressed during sepsis and potentiated by landiolol. (*N* = 5–6 per group). *CLP* cecal ligation and puncture, *CLP* + *Lnd* CLP plus landiolol, *Nb of genes* number of genes

In the males, the comparison of DEGs in the sham and CLP groups revealed 2850 DEGs (1259 up-regulated genes and 1591 down-regulated genes). In the females, only 1267 DEGs were found (506 up-regulated genes and 761 down-regulated genes). Males and females shared 803 DEGs, including 328 up-regulated genes (26% of male genes and 64% of female genes) and 475 down-regulated genes (30% of male genes and 62% of female genes) (Fig. 1a).

In the CLP plus landiolol group, 1021 DEGs and 83 DEGs were differently regulated in males and females, representing 603 and 71 up-regulated genes and 418 and 12 down-regulated genes (Fig. 1b). Further, 60 and 86% of DEGs were up-regulated in males and females, respectively. The lists of DEGs are shown in the Additional file 1.

To assess CLP-related functional annotations, we used biological processes, gene ontology terms and KEGG pathways using the DAVID database for the 2850 and the 1267 DEGs from males and females, respectively. For up-regulated genes, the most significant biological processes were associated with inflammatory and immune responses, apoptosis, metabolism, oxidative stress, muscle cell proliferation, protein kinase activity and the development of primary sexual features. For down-regulated genes, the biological processes were related to mitochondrion, muscular cell organisation (organelles, myocyte membrane and contractile proteins), muscle contraction, blood circulation, ionic transport and phosphoinositide-3-kinase/AKT (PI3K/AKT) signalling pathway. These changes in gene expression were predominantly found in males (Fig. 1c, Additional file 3: Table S1). For example, in the male CLP group, the transcript levels of the Janus kinase/signal transducer and activator of transcription (JAK/STAT) pathway interleukin (IL) 10, IL6R, JAK, STAT and cyclin-dependent kinase inhibitor 1A (p21) were increased, while CycD (CCND1) and B cell CLL/Lymphoma 2 (BCL2) were decreased. In the CLP female group, IL10, p21 and BCL2 were not regulated (Additional file 1).

In males, landiolol down-regulated genes of the biological processes were related to up-regulated genes of the inflammatory response, apoptosis, metabolism and oxidative stress. In contrast, immunity-related signalling further increased after landiolol. Genes associated with other biological processes (i.e. ionic transport, cell differentiation and cytoskeleton organisation), which were down-regulated in the male CLP group, were up-regulated after landiolol. Due to the low number of DEGs in the female rats, neither up- nor down-regulated genes were associated with significant biological processes (Fig. 1d, Additional file 3: Table S2).

### DEGs in the CLP group with reverted expression in the CLP plus landiolol group

To better understand the mechanisms resulting in cardiac performance improvement with landiolol, we assessed genes with a V-form expression, meaning that genes up- or down-regulated in sepsis compared to sham were back-regulated after landiolol administration.

**Fig. 2:**
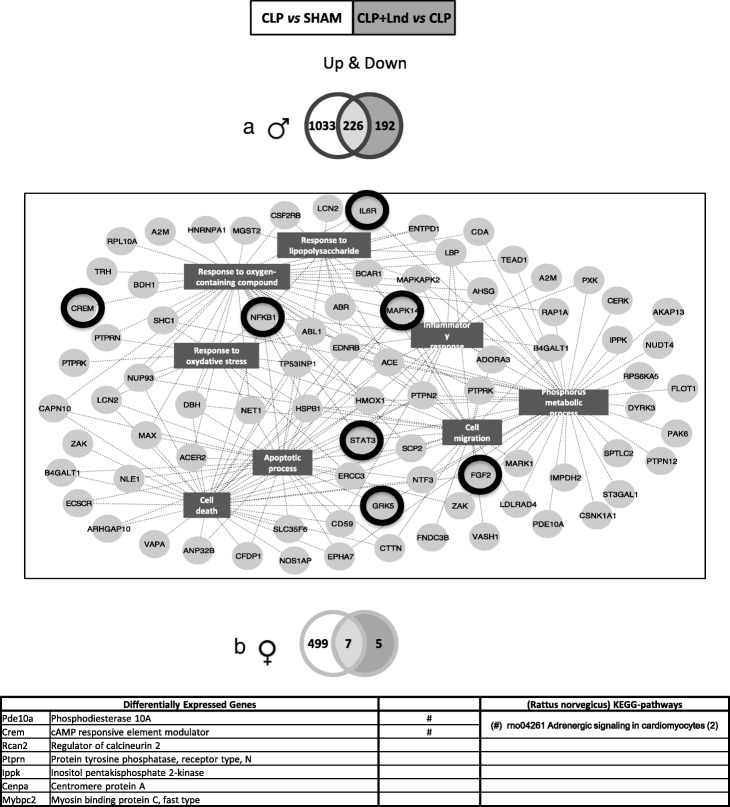
Biological processes associated with up-regulated genes after cecal ligation and puncture and back-regulated by landiolol infusion. **a** Venn diagram associated with the 1259 up-regulated genes in CLP group and the 418 down-regulated genes in CLP plus landiolol group in males. The 226 common genes represent regulated genes with a characteristic V form, up-regulated in sepsis and back-regulated by landiolol infusion. From these 226 common genes, a gene ontology analysis with the DAVID Database revealed significant biological processes (P-Benjamini < 0.05). A gene network diagram was performed using Cytoscape integrating the 226 genes (light grey circles) with their associated biological processes (dark grey rectangles). Genes associated with the JAK/STAT pathway, IL6R and STAT, with the PI3K/AKT and focal adhesion pathways, FGF2 and NFκB1, and with the mitogen-activated protein kinase and tumour necrosis factor-α pathways, cyclic adenosine monophosphate responsive element modulator and MAPK14 and GRK5, are surrounded by a black circle. **b** Venn diagram associated with the 506 up-regulated genes in CLP group and the 12 down-regulated genes in CLP plus landiolol group in females. Note that only 12 genes were down-regulated in CLP plus landiolol condition and that only seven of them were back-regulated. No significant biological processes were found associated to these seven genes presented in the table; two genes are members of the adrenergic signalling in the cardiomyocyte KEGG-pathway and the others are isolated genes. *CLP* cecal ligation and puncture, *CLP* + *Lnd* CLP plus landiolol

In the male CLP group, 1259 genes were up-regulated. Landiolol down-regulated 418 genes. A V form was found for 226 genes (Additional file 2). For an overview of their biological activities, significant biological processes were identified and an integrative network was generated using Cytoscape (Fig. 2a). Landiolol reversed genes associated with inflammation, oxidative stress, oxygen-containing compound, and lipopolysaccharide responses, phosphorus metabolic and apoptotic processes, cell death and migration. For example, genes associated with the JAK/STAT pathway, IL6R and STAT, with the PI3K/AKT and focal adhesion pathways, fibroblast growth factor 2 (FGF2) and nuclear factor kappa B subunit 1 (NFκB1), and with the mitogen-activated protein kinase (MAPK) and tumour necrosis factor-α (TNF-α) pathways, cyclic adenosine monophosphate (cAMP) responsive element modulator and MAPK14, were back-regulated by landiolol infusion (Fig. 2a). Expression levels of the G protein-coupled receptor kinase 5 (GRK5) was also restored almost to the sham group level by landiolol infusion (Fig. 2a). In females, only 12 genes were down-regulated in the CLP plus landiolol group and seven genes displayed a V form (Fig. 2b). None of the genes were associated with significant biological processes. Two genes, Phosphodiesterase 10A (PDE10A) and cAMP-responsive element modulator, were associated with the adrenergic signalling KEGG pathway.

**Fig. 3:**
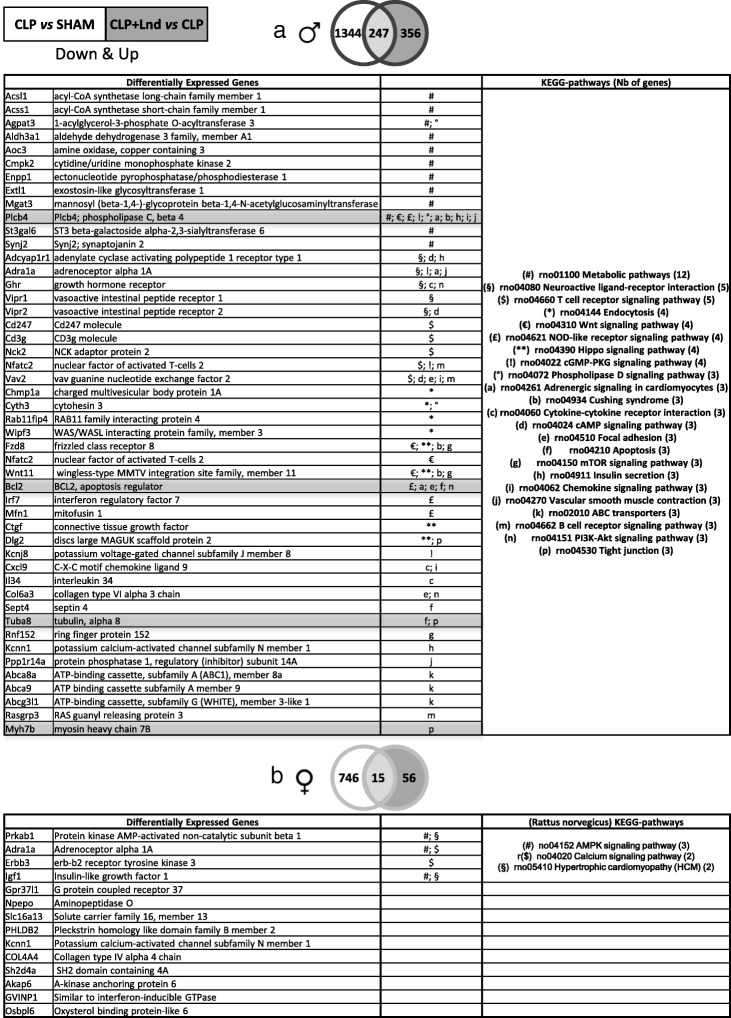
KEGG-pathways associated with down-regulated genes after cecal ligation and puncture and back-regulated by landiolol infusion. **a** Venn diagram associated with the 1591 down-regulated genes in CLP group and the 603 up-regulated genes in CLP plus landiolol group in males. The 247 common genes represent regulated genes with a characteristic V form, down-regulated in sepsis and back-regulated by landiolol infusion. No significant biological processes were found associated to these 247 common genes. The table presents KEGG-pathways in which at least three of these 247 common genes are involved. Genes associated with the JAK/STAT pathway, BCL2 and AOX, and with the adrenergic pathway, PLCB4, TUBA8 and MYH7B, are highlighted in grey. **b** Venn diagram associated with the 761 down-regulated genes in CLP group and the 71 up-regulated genes in CLP plus landiolol group in females. Note that only 71 genes were up-regulated in the CLP plus landiolol group and that only 15 of them were back-regulated. No biological processes were found associated to these 15 common genes presented in the table; four genes are members of specific KEGG-pathways and the others are isolated genes. *CLP* cecal ligation and puncture, *CLP* + *Lnd* CLP plus landiolol

Regarding the CLP down-regulated genes, no associated biological processes were found for genes with a V form either in males or in females. A list of the 247 genes found in males is presented in Additional file 2. The KEGG pathways associated with these DEGs were classified according to the number of genes found in each pathway (Fig. 3). The metabolic pathway was the most represented in males. Other pathways, for example T cell receptor signalling, NOD-like receptor signalling, Wnt signalling, Hippo signalling, adrenergic signalling, cAMP signalling, cGMP-dependant protein kinase G (PKG) signalling and PI3K-Akt signalling, were found only in males (Fig. 3a). For example, genes associated with the JAK/STAT pathway, BCL2 and aldehyde oxidase 3 (AOX), or with the adrenergic pathway, phospholipase C beta 4 (PLCB4), tubulin alpha 8 (TUBA8) and myosin heavy chain 7B cardiac muscle beta (MYH7B), were back-regulated by landiolol infusion (Fig. 3a and Additional file 3: Table S2). In females, only the AMP-activated protein kinase (AMPK) signalling pathway was found associated with three significant DEGs (Fig. 3b).

## Discussion

In this study, we identified biological processes and signalling pathways associated with the cardiac response to landiolol in septic male and female rats. This is the first genome-wide analysis of the sex-mediated response to a β-blocker in sepsis-induced myocardial dysfunction. Our results highlight differences between males and females, during sepsis and after landiolol administration. Several biological processes were found only in males. In contrast, effects were limited in females. These results support our previous findings obtained by MRI studies, showing decreased indexed stroke volume (SVi), cardiac index (CI) and indexed end-diastolic volume (EDVi) after CLP in males, whereas only CI and EDVi decreased in females. Landiolol improved cardiac performance in the septic males by increasing SVi. In the septic females, landiolol reduced left ventricular ejection fraction (LVEF) and systolic wall thickening (sWtn). Blood pressure remained constant in males but decreased in females after landiolol [26].

A higher number of deregulated genes were found in males, affecting the inflammatory response, the innate immune response and the gonad development, which were enriched by up-regulated genes. Biological processes enriched by down-regulated genes were the organisation of the extracellular matrix, muscle cell differentiation, muscle tissue development, sarcolemma and T-tubules, muscle and cardiac contraction, contractile fibres and sarcomeres, potassium transmembrane transport, positive regulation of calcium transport and the PI3K/AKT signalling pathway. In summary, inflammatory response, apoptosis, metabolism, oxidative stress and calcium ion transport were down-regulated specifically in males. In females, very few sepsis-induced DEGs and no significant biological processes were detected after landiolol administration.

Our results are in line with findings on the human septic cardiomyopathy. Down-regulation of cardiac mitochondrial genes, in addition to multiple alterations in sarcomeric genes and genes that maintain the structural integrity of the sarcolemma, were described [31]. Matkovich et al. hypothesised that the cardiac response to sepsis was coordinated in a ‘programmatic’ mode, as described in myocardial hibernation [31].

In male rats, CLP had profound effects on the expression of genes involved in JAK/STAT, PI3K and focal adhesion pathways. Moreover, CLP up-regulated transcripts encoding for p53-dependent cell-cycle arrest (p21 and stromal antigen 1 (STAG1)), toll-like receptor 1 (TLR1) and myeloid differentiation primary response 88 (MyD88). These changes were not described in females. These results support findings of the pronounced myocardial dysfunction in male rats, as compared to female rats [26]. They are in line with data published by Rudiger et al. in which these pathways were associated with poor outcome [17]. An increased systemic inflammatory response through circulating myocardial depressant factors, including nitric oxide (NO), may compromise myocardial function [17]. The level of cyclic guanosine monophosphate (cGMP), generated by soluble guanylyl cyclase (GC) and regulated by NO, played opposite roles in males and females, being protective in males and deleterious in females [29]. In our model, the endothelial NO synthase (eNOS) protein level was increased [26] and the transcript of the guanylate cyclase 1 soluble subunit alpha 2 (GUCY1A2) was decreased in the male CLP group. Interestingly, landiolol reversed the expression of most of the transcripts of the aforementioned signalling pathways and the increase in eNOS expression only in males [26].

Sex-related differences detected in transcript abundance of the β-adrenergic signalling and calcium cycling pathways were associated with reduced survival [17]. These changes may lead to a reduced L-type calcium current [32], and a high cytosolic Ca^2+^ level, leading to failure of diastolic relaxation and low sarcoplasmic reticulum Ca^2+^ levels, which affect systolic contraction as previously observed in septic cardiomyopathy [33]. A decrease of SERCA3 was found specifically in the male CLP group, corresponding to the significant decrease of the indexed stroke volume in the MRI [26]. Sex differences in the mechanisms of the contractile function and in the response to the β-adrenergic stimulation were previously described [34, 35]. In lipopolysaccharide-induced cardiomyopathy, sarcomere shortening depression occurred with different regulations of cellular calcium transients and SERCA in male and female mice [27]. Deficits in mechanisms downstream of cellular calcium transients, possibly a decrease in myofilament sensitivity for calcium following troponin I hyperphosphorylation [36], can explain this difference. Moreover, oestrogen may modulate the properties of the ryanodine receptor [37] and the interplay between oestrogen receptors and the β-adrenergic receptors could affect Ca^2+^-handling proteins and the phosphoinositide-3-kinase-AKT (PI3K-AKT) pathway in a sex-dependent manner [38].

Finally, data on sex dimorphism in the response to landiolol during septic cardiomyopathy should be considered in patient studies, in conjunction with an investigation of inflammation attenuation, improved outcome and heart rate control [39–42].

Our study has some limitations. The ex vivo assessment of cardiac function at the end of the protocol might have altered the expression of transcripts. However, the procedure was similar for all the groups and we used a differential analysis to minimise the effect of this procedure. Our results are consistent with those of in vivo studies in which this ex vivo procedure is absent [17], and with those published in humans using the same type of differential analysis [31]. Another limitation is the absence of calculations to determine the sample-size required. However, Allison et al. examined key components of microarray analysis and indicated that, for common designs in which two groups of cases are evaluated for differential expression, a minimum of five biological replicates per group should be analysed [30]. In our study, we had 5–6 biological replicates for each group and chose the 2-class SAM analysis to detect significant variation in gene expression between groups (CLP group vs sham group and CLP plus landiolol group vs CLP group). The number of altered genes does not predict the severity of the organ dysfunction, and changes in gene expression do not necessarily reflect changes in the level of protein expression. Our results need to be confirmed at the protein level. In addition, we focused our transcriptomic analyses on cardiac tissue. However, it is reasonable to speculate that each organ has a specific transcriptomic response to infection, as reported previously for the liver [43]. The timing of each experiment may affect the findings [44]. In our study, changes in gene expression were analysed 20 h after CLP. This can explain differences to previous experiments in which measures were performed at 6 h post-insult [17] and 48 h post-insult [45]. Another limitation is that despite fluid administration at 10 ml kg^−1^ h^−1^, we cannot rule out hypovolemia related to capillary leak in the CLP animals. Rudiger et al. attenuated the decline in stroke volume and left ventricular end-diastolic volume by the use of fluid resuscitation consisting of a 1:1 solution of 6% hetastarch and an additional 25 ml kg^−1^ body weight of fluid boluses at 6 h and 10 ml kg^−1^ at 24 h [17]. In addition, the response to the opioid buprenorphine, used for pain control, might have affected gene expression in a sex-dependent manner. Doyle et al. described sex differences in morphine metabolism and associated circulatory cytokines levels [46]. We have established our experimental conditions to prevent septic shock in animals. We did not use antibiotics and no animals died during the experiments. Our results may therefore differ from those obtained in humans or in some animals resuscitated with both fluids and antibiotics. Moreover, most intensive care unit patients receive sedation including opioids [47].

## Conclusion

In a CLP-induced animal model of sepsis, the regulation of myocardial transcripts in male rats differed from that in female rats. We found common functional annotations induced by sepsis in both sexes, but many processes associated with the development, differentiation, organisation and function of cardiac muscle cells were deregulated only in males. These results were in agreement with the pathological status of the animals and demonstrated that sepsis-related changes in gene transcription were more pronounced in males than in females. Landiolol had diverse effects on biological processes in male and female rats. After landiolol administration, the expression of genes related to different biological processes was reversed in males. This possibly explains the improvement in the cardiac function of the male animals in the landiolol-treated group. No significant functional annotation was found in females in the landiolol-treated group. Only few genes associated with adrenergic, protein kinase AMP-activated catalytic subunit alpha, calcium and hypertrophic signalisation were differentially expressed and may explain the deleterious effects on cardiac function observed after landiolol in females.

## Additional files

Additional file 1: Lists of differentially expressed genes in male and female hearts after cecal ligation and puncture and landiolol. Lists of significantly up- and down-regulated genes, obtained after 2-class SAM, in CLP group vs sham group (FDR < 0,01) and in CLP plus landiolol group vs CLP (FDR < 0,05) in males and females. Common genes between males and females are noted in blue. (XLSX 211 kb)

Additional file 2: Lists of differentially expressed genes with a characteristic V form in male. Lists of the significantly up- and down-regulated genes in sepsis and back-regulated by landiolol infusion in males. (XLSX 41 kb)

Additional file 3: **Table S1.** GO annotations for differentially expressed genes in male and female hearts after cecal ligation and puncture. Gene ontology analysis realized with the DAVID Database is reported in the table. The most significant biological processes (BP), cellular components (CC) and KEGG-pathways (KEGG) GO terms for up- and down-regulated genes are indicated for CLP vs sham groups in males and females. For all the processes, the number of genes deregulated in males is much greater than in females. *N. of genes = Number of genes; NS = not significant; ø = not found.* **Table S2.** GO annotations for differentially expressed genes in male and female hearts after landiolol administration. Gene ontology analysis realized with the DAVID Database is reported in the table. Significant biological processes (BP) and KEGG-pathway (KEGG) GO terms for up- and down-regulated genes are indicated for CLP plus landiolol vs CLP groups in males and females. For all the processes, the number of genes deregulated in males is much greater than in females and significant biological processes were totally absent for females after landiolol infusion. *N. of genes = Number of genes; NS = not significant; ø = not found*. (PPTX 51 kb)

## Abbreviations

AR: Adrenergic receptor
BCL2: B-cell CLL/lymphoma 2
cAMP: Cyclic adenosine monophosphate
CI: Cardiac index
CLP: Cecal ligation and puncture
DAVID: Database for Annotation, Visualization and Integrated Discovery
DEG: Differentially expressed gene
EDVi: End-diastolic volume index
FDR: False discovery rate
GRK: G protein-coupled receptor kinase 5
IL: Interleukin
JAK/STAT: Janus kinase/signal transducer and activator of transcription
KEGG: Kyoto Encyclopedia of Genes and Genomes
MAPK: Mitogen-activated protein kinase
MRI: Magnetic resonance imaging
MYH7B: Myosin heavy chain 7B cardiac muscle beta
NO: Nitric oxide
NOS: Nitric oxide synthase
p21: Cyclin-dependent kinase inhibitor 1A
PGC-1α: Peroxisome proliferator-activated receptor gamma, coactivator 1 alpha
PI3K-AKT: Phosphoinositide-3-kinase-AKT
PKA: Protein kinase cAMP-activated catalytic subunit alpha
PP2: Protein phosphatase 2 catalytic subunit alpha
SAM: Significance analysis of microarrays
SERCA: Sarcoplasmic reticulum calcium ATPase
SVi: Stroke volume index
TNF-α: Tumour necrosis factor-α
TUBA: Tubulin alpha 8
ΔCai: Cellular calcium transients
