# Supplementary material for: Effect of landiolol on sex-related transcriptomic changes in the myocardium during sepsis
Source: Intensive Care Med Exp. 2019 Aug 19;7:50. doi: 10.1186/s40635-019-0263-0 (PMC6701793; doi:10.1186/s40635-019-0263-0)
Supplement: Supplementary file 3 — Table S1. GO annotations for differentially expressed genes in male and female hearts after cecal ligation and puncture. Gene ontology analysis realized with the DAVID Database is reported in the table. The most significant biological processes (BP), cellular components (CC) and KEGG-pathways (KEGG) GO terms for up- and down-regulated genes are indicated for CLP vs sham groups in males and females. For all the processes, the number of genes deregulated in males is much greater than in females. N. of genes = Number of genes; NS = not significant; ø = not found. Table S2. GO annotations for differentially expressed genes in male and female hearts after landiolol administration. Gene ontology analysis realized with the DAVID Database is reported in the table. Significant biological processes (BP) and KEGG-pathway (KEGG) GO terms for up- and down-regulated genes are indicated for CLP plus landiolol vs CLP groups in males and females. For all the processes, the number of genes deregulated in males is much greater than in females and significant biological processes were totally absent for females after landiolol infusion. N. of genes = Number of genes; NS = not significant; ø = not found. (PPTX 51 kb) [file 40635_2019_263_MOESM3_ESM.pptx]

## Slide 1
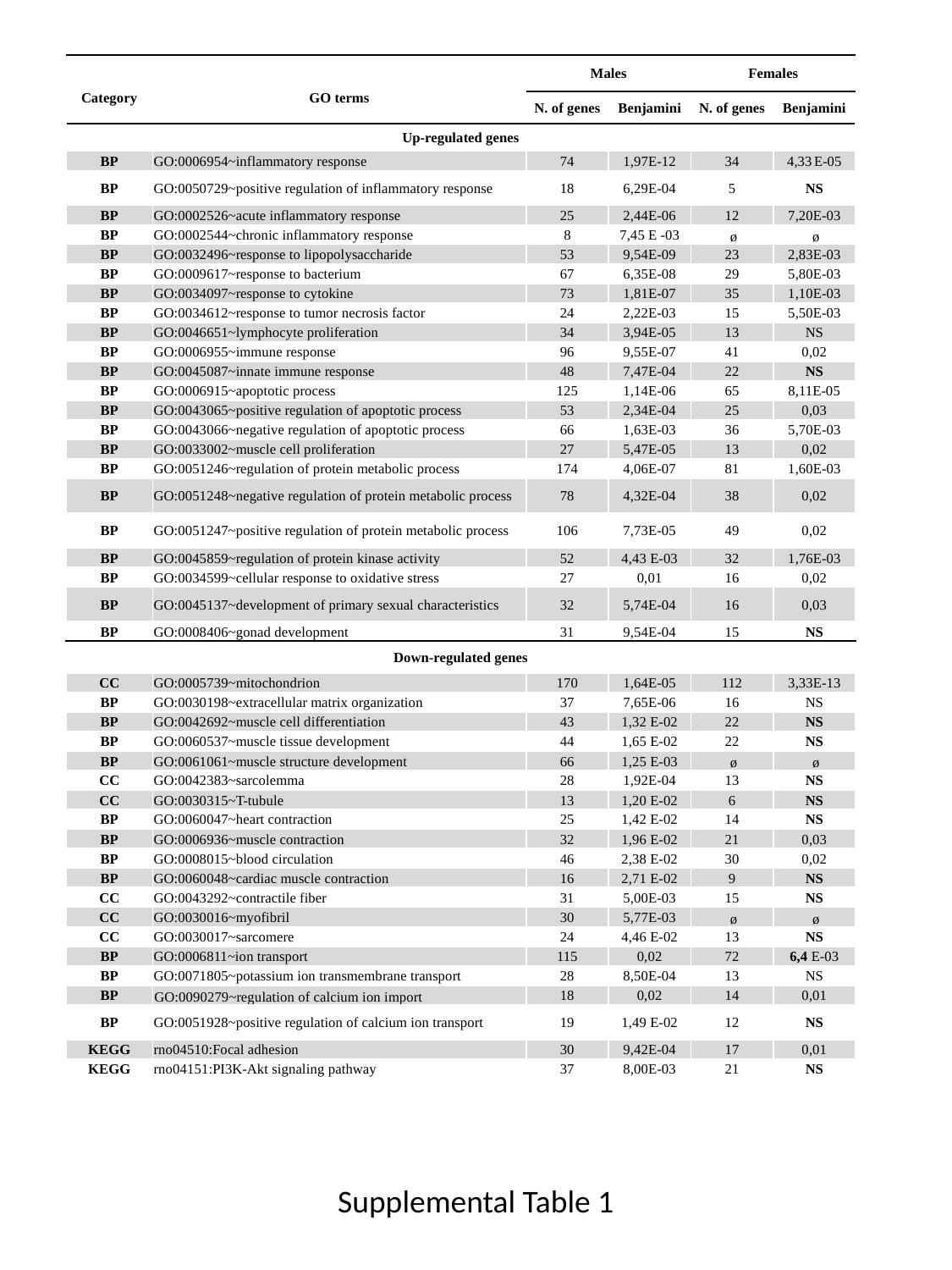

| Category | GO terms | Males | | Females | |
| --- | --- | --- | --- | --- | --- |
| | | N. of genes | Benjamini | N. of genes | Benjamini |
| Up-regulated genes | | | | | |
| BP | GO:0006954~inflammatory response | 74 | 1,97E-12 | 34 | 4,33 E-05 |
| BP | GO:0050729~positive regulation of inflammatory response | 18 | 6,29E-04 | 5 | NS |
| BP | GO:0002526~acute inflammatory response | 25 | 2,44E-06 | 12 | 7,20E-03 |
| BP | GO:0002544~chronic inflammatory response | 8 | 7,45 E -03 | ø | ø |
| BP | GO:0032496~response to lipopolysaccharide | 53 | 9,54E-09 | 23 | 2,83E-03 |
| BP | GO:0009617~response to bacterium | 67 | 6,35E-08 | 29 | 5,80E-03 |
| BP | GO:0034097~response to cytokine | 73 | 1,81E-07 | 35 | 1,10E-03 |
| BP | GO:0034612~response to tumor necrosis factor | 24 | 2,22E-03 | 15 | 5,50E-03 |
| BP | GO:0046651~lymphocyte proliferation | 34 | 3,94E-05 | 13 | NS |
| BP | GO:0006955~immune response | 96 | 9,55E-07 | 41 | 0,02 |
| BP | GO:0045087~innate immune response | 48 | 7,47E-04 | 22 | NS |
| BP | GO:0006915~apoptotic process | 125 | 1,14E-06 | 65 | 8,11E-05 |
| BP | GO:0043065~positive regulation of apoptotic process | 53 | 2,34E-04 | 25 | 0,03 |
| BP | GO:0043066~negative regulation of apoptotic process | 66 | 1,63E-03 | 36 | 5,70E-03 |
| BP | GO:0033002~muscle cell proliferation | 27 | 5,47E-05 | 13 | 0,02 |
| BP | GO:0051246~regulation of protein metabolic process | 174 | 4,06E-07 | 81 | 1,60E-03 |
| BP | GO:0051248~negative regulation of protein metabolic process | 78 | 4,32E-04 | 38 | 0,02 |
| BP | GO:0051247~positive regulation of protein metabolic process | 106 | 7,73E-05 | 49 | 0,02 |
| BP | GO:0045859~regulation of protein kinase activity | 52 | 4,43 E-03 | 32 | 1,76E-03 |
| BP | GO:0034599~cellular response to oxidative stress | 27 | 0,01 | 16 | 0,02 |
| BP | GO:0045137~development of primary sexual characteristics | 32 | 5,74E-04 | 16 | 0,03 |
| BP | GO:0008406~gonad development | 31 | 9,54E-04 | 15 | NS |
| Down-regulated genes | | | | | |
| CC | GO:0005739~mitochondrion | 170 | 1,64E-05 | 112 | 3,33E-13 |
| BP | GO:0030198~extracellular matrix organization | 37 | 7,65E-06 | 16 | NS |
| BP | GO:0042692~muscle cell differentiation | 43 | 1,32 E-02 | 22 | NS |
| BP | GO:0060537~muscle tissue development | 44 | 1,65 E-02 | 22 | NS |
| BP | GO:0061061~muscle structure development | 66 | 1,25 E-03 | ø | ø |
| CC | GO:0042383~sarcolemma | 28 | 1,92E-04 | 13 | NS |
| CC | GO:0030315~T-tubule | 13 | 1,20 E-02 | 6 | NS |
| BP | GO:0060047~heart contraction | 25 | 1,42 E-02 | 14 | NS |
| BP | GO:0006936~muscle contraction | 32 | 1,96 E-02 | 21 | 0,03 |
| BP | GO:0008015~blood circulation | 46 | 2,38 E-02 | 30 | 0,02 |
| BP | GO:0060048~cardiac muscle contraction | 16 | 2,71 E-02 | 9 | NS |
| CC | GO:0043292~contractile fiber | 31 | 5,00E-03 | 15 | NS |
| CC | GO:0030016~myofibril | 30 | 5,77E-03 | ø | ø |
| CC | GO:0030017~sarcomere | 24 | 4,46 E-02 | 13 | NS |
| BP | GO:0006811~ion transport | 115 | 0,02 | 72 | 6,4 E-03 |
| BP | GO:0071805~potassium ion transmembrane transport | 28 | 8,50E-04 | 13 | NS |
| BP | GO:0090279~regulation of calcium ion import | 18 | 0,02 | 14 | 0,01 |
| BP | GO:0051928~positive regulation of calcium ion transport | 19 | 1,49 E-02 | 12 | NS |
| KEGG | rno04510:Focal adhesion | 30 | 9,42E-04 | 17 | 0,01 |
| KEGG | rno04151:PI3K-Akt signaling pathway | 37 | 8,00E-03 | 21 | NS |
Supplemental Table 1

## Slide 2
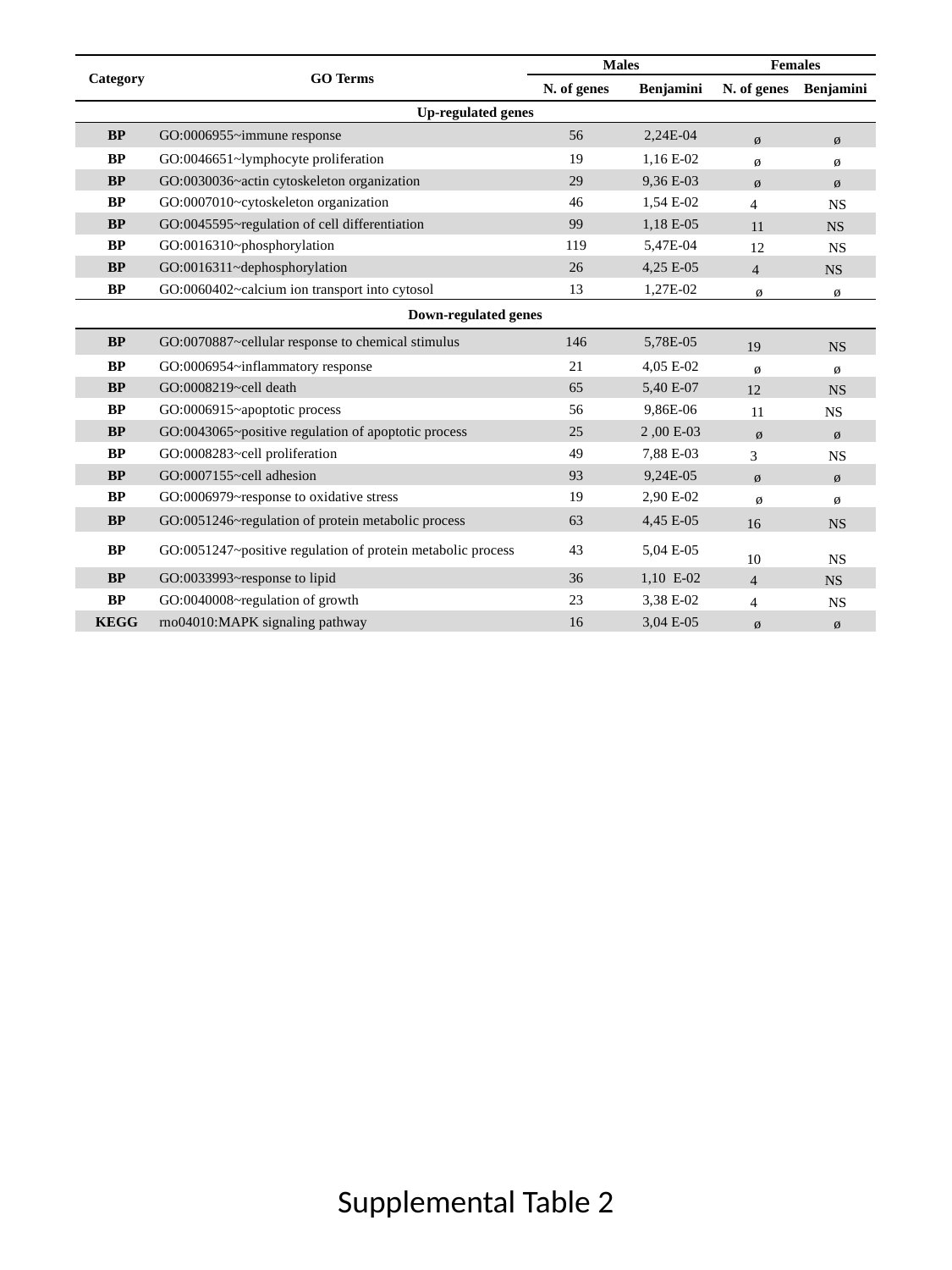

| Category | GO Terms | Males | | Females | |
| --- | --- | --- | --- | --- | --- |
| | | N. of genes | Benjamini | N. of genes | Benjamini |
| Up-regulated genes | | | | | |
| BP | GO:0006955~immune response | 56 | 2,24E-04 | ø | ø |
| BP | GO:0046651~lymphocyte proliferation | 19 | 1,16 E-02 | ø | ø |
| BP | GO:0030036~actin cytoskeleton organization | 29 | 9,36 E-03 | ø | ø |
| BP | GO:0007010~cytoskeleton organization | 46 | 1,54 E-02 | 4 | NS |
| BP | GO:0045595~regulation of cell differentiation | 99 | 1,18 E-05 | 11 | NS |
| BP | GO:0016310~phosphorylation | 119 | 5,47E-04 | 12 | NS |
| BP | GO:0016311~dephosphorylation | 26 | 4,25 E-05 | 4 | NS |
| BP | GO:0060402~calcium ion transport into cytosol | 13 | 1,27E-02 | ø | ø |
| Down-regulated genes | | | | | |
| BP | GO:0070887~cellular response to chemical stimulus | 146 | 5,78E-05 | 19 | NS |
| BP | GO:0006954~inflammatory response | 21 | 4,05 E-02 | ø | ø |
| BP | GO:0008219~cell death | 65 | 5,40 E-07 | 12 | NS |
| BP | GO:0006915~apoptotic process | 56 | 9,86E-06 | 11 | NS |
| BP | GO:0043065~positive regulation of apoptotic process | 25 | 2 ,00 E-03 | ø | ø |
| BP | GO:0008283~cell proliferation | 49 | 7,88 E-03 | 3 | NS |
| BP | GO:0007155~cell adhesion | 93 | 9,24E-05 | ø | ø |
| BP | GO:0006979~response to oxidative stress | 19 | 2,90 E-02 | ø | ø |
| BP | GO:0051246~regulation of protein metabolic process | 63 | 4,45 E-05 | 16 | NS |
| BP | GO:0051247~positive regulation of protein metabolic process | 43 | 5,04 E-05 | 10 | NS |
| BP | GO:0033993~response to lipid | 36 | 1,10 E-02 | 4 | NS |
| BP | GO:0040008~regulation of growth | 23 | 3,38 E-02 | 4 | NS |
| KEGG | rno04010:MAPK signaling pathway | 16 | 3,04 E-05 | ø | ø |
Supplemental Table 2
